# Transfer-Learning-Based Approach for the Diagnosis of Lung Diseases from Chest X-ray Images

**DOI:** 10.3390/e24030313

**Published:** 2022-02-22

**Authors:** Rong Fan, Shengrong Bu

**Affiliations:** 1 School of Engineering and Applied Science, University of Pennsylvania, Philadelphia, PA 19104, USA; rongfan@seas.upenn.edu; 2 Department of Engineering, Brock University, St. Catharines, ON L2S 3A1, Canada

**Keywords:** transfer learning, deep learning, pretrained neural networks, chest X-ray images, lung diseases

## Abstract

Using chest X-ray images is one of the least expensive and easiest ways to diagnose patients who suffer from lung diseases such as pneumonia and bronchitis. Inspired by existing work, a deep learning model is proposed to classify chest X-ray images into 14 lung-related pathological conditions. However, small datasets are not sufficient to train the deep learning model. Two methods were used to tackle this: (1) transfer learning based on two pretrained neural networks, DenseNet and ResNet, was employed; (2) data were preprocessed, including checking data leakage, handling class imbalance, and performing data augmentation, before feeding the neural network. The proposed model was evaluated according to the classification accuracy and receiver operating characteristic (ROC) curves, as well as visualized by class activation maps. DenseNet121 and ResNet50 were used in the simulations, and the results showed that the model trained by DenseNet121 had better accuracy than that trained by ResNet50.

## 1. Introduction

Many people suffer from lung diseases such as pneumonia and emphysema every year. Chest X-ray images are one of the most widely used and low-cost diagnose tools for lung diseases [1]. However, since there might be more than one pathology to be detected from chest X-rays for a disease [2], diagnosing by doctors could be challenging sometimes. Computer-aided diagnosis for various diseases has been researched to improve the efficiency and accuracy of the diagnosis [3]. Various deep learning methods [4] for medical image classification have the potential of predicting and diagnosing diseases even more accurately than the average radiologist [5].

Since the global corona virus pandemic, researchers have developed methods to analyze radiographic chest images more efficiently to make the diagnosis of COVID-19 easier. Heidari et al. developed a novel deep learning model to detect non-pneumonia, non-COVID-19-infected pneumonia and COVID-19-infected pneumonia [6]. In [7], the authors presented a deep learning approach to realize the diagnosis of pulmonary hypertension by analyzing chest radiographs and compared the performance of ResNet50, Xception, and Inception V3. Yu et al. built a multi-task deep learning network consisting of an extraction architecture and three different routes for various functions by using chest X-rays from peripherally inserted central catheters [8]. Jaiswal et al. realized the localization and identification of pneumonia in chest X-ray images using a deep learning model derived from mask-RCNN [9]. In [5], a modified AlexNet with many handcrafted features was proposed to detect whether the chest X-ray images were in the normal or in the pneumonia class.

However, the medical image dataset could be too small to be used to train a neural network since the images have to be labeled by professionals. Transfer learning originated from terms such as knowledge transfer or inductive transfer in 1995 [10], and later, in 2005, it was defined as the technique of applying knowledge and skills learned in previous tasks to novel tasks [11]. Since then, many studies have employed transfer learning on small medical datasets and trained neural networks to realize image recognition and classification. Minaee et al. applied transfer learning to process chest X-ray images for the detection of COVID-19, and DenseNet121, ResNet18, ResNet50, and SqueezeNet were utilized as the pre-trained networks [12]. In [13], the advantages and challenges of deep transfer learning were studied. Ravishankar et al. realized ultrasound kidney images’ detection using transfer learning [14]. A deep convolutional neural network (DCNN) was proposed to study the advantages of transfer learning in medicine [15]. Subspace-based techniques, such as in [16], can be used together with transfer learning to increase the accuracy when the dataset is small.

Class imbalance is a common challenging related to medical image diagnosis [17], since the amount of positive data and negative ones in each class might not be equivalent. In this kind of application, the rare or minor occurrences are much more important than the majority classes [18]. As a result, the contributions of the loss for these two kinds of data are not the same, and the small data size of some class will affect the overall training performance. Various methods could be used to handle imbalanced datasets, including setting appropriate class weights for the model and random under-sampling and over-sampling.

In this paper, a transfer learning method is proposed to classify 14 lung-related pathologies using frontal-view chest X-ray images. The contributions of this paper are as follows:

- We built image classification models using pretrained networks;
- We preprocessed the data including data augmentation of the ChestX-ray8 dataset and dealt with the class imbalance problem;
- We trained, validated, and tested the model using pretrained networks and compared the performance of each model using the ROC curves. We visualized the classification decision using Grad-CAM.

The structure of this paper is as follows. The methods and principles with respect to transfer learning, data augmentation, evaluation, and visualization are presented in Section 2. Section 3 then presents the experimental process and results. Finally, the conclusion of this paper is drawn in Section 4.

## 2. Proposed Transfer Learning Method

In our work, transfer learning was used for the chest X-ray image classification task. Transfer learning is an effective method in the image processing domain that can take advantage of well-developed models to solve new tasks [19]. There are two main ways to utilize pretrained networks in transfer learning: First, a pretrained model can be used as the feature extractor for the new dataset. Once the features are extracted, added layers such as a linear classifier can be trained for the new task. Second, the whole or some part of the pretrained network will be fine-tuned for the new classification task. Thus, the weights of the pretrained model are considered as the initial values and will be updated during the training process. In our work, the first method was used since the dataset was small and the computing power was limited. Two networks, i.e., DenseNet121 and ResNet50, were used as the base models for transfer learning. In the following, the principle of transfer learning, the framework of the networks, and the measures for the evaluation are discussed.

### 2.1. Transfer Learning with a Data Augmentation Approach

Two pretrained networks were employed as the training models in this project. The first one is called ResNet50, which won the first prize in the 2015 ImageNet competition. This model uses a shortcut connection, which is the basis of a residual network, and the connection ensures that the feature of one preceding layer is the input of the later layers, skipping some of the layers. Therefore, any layer in this framework has information from the preceding layers. The design overcomes the problem of learning rate reduction and invariant classification accuracy as a result of a deeper network. The second one is DenseNet121, which was the winner of the 2017 ImageNet competition and has been widely applied in deep learning. DenseNet consists of DenseBlock layers, each of which receives additional inputs from all preceding layers and transition layers. Additional inputs from all preceding layers together with the feature maps of the current layer are all passed on to other subsequent layers, and thus, the shortcuts of all the former layers and the latter layer are built densely. For comparison, the traditional CNN with *l* layers has *l* connections between adjacent layers, whereas DenseNet has l(l+1)/2 layers in total because of its shortcut feature [6]. Thus, the learned features could be reused and the network has less channels as a result of the collective knowledge feature of each layer. Besides, this also leads to better performance under the conditions of fewer parameters and little computing cost. It also has some other advantages such as vanishing gradient problem mitigation and parameter reduction. In contrast, since ResNet only has shortcuts between the former layer and the latter layer, and DenseNet has demonstrated better performance. Due to the aforementioned reasons, DenseNet is much deeper than ResNet and has more than 100 layers, and the training process could be more effective and the accuracy improved.

One basic problem of deep learning is the opposition of optimization and generalization [20]. Optimization is the learning process that adjusts the model to obtain the best performance, while generalization is the performance of the model on the testing of new data. The goal of learning is to realize a satisfactory generation, but this cannot be controlled, so the models are always adjusted based on the training data. When the training process begins, the generalization can become worse after a number of iterations, which means the model is overfitting, and this is a common problem in training neural networks. Among various methods used to prevent the neural networks from overfitting, data augmentation is the most effective one and is widely used in computer vision, especially when the dataset is small. In *Keras*, data augmentation can be realized by using the *ImageDataGenerator* class and transforming the image parameters randomly. Some commonly adjusted parameters include the following: rotation_range is the rotation range of the image; width_shift and height_shift are the range of shifting in the horizontal and vertical direction, respectively; horizontal_flip is the flip ratio; sheer_range is the random sheer angle of the image.

### 2.2. Evaluation Methods

The performance of the network needs to be evaluated after testing. Accuracy and receiver operating characteristic (ROC) curves with the AUCROC were used as the metrics for the evaluation. Accuracy shows the general performance of all testing images, and the ROC curves with the AUCROC indicate the classification performance for each label.

The classification task in our project was a multi-task classification because one image might correspond to more than one pathological condition. Therefore, the *Accuracy* can be calculated as follows, since there are 14 pathological conditions:

(1) $$Accuracy=\frac{\mathrm{sumof}\mathrm{truly}\mathrm{predicted}\mathrm{labels}}{14*(\#\mathrm{of}\mathrm{testing}\mathrm{images})}$$

The accurately predicted labels for all *testing images* were considered together instead of calculating the accuracy of each image and then averaging them. The *sum of the truly predicted labels* was calculated by first finding the number of truly predicted images for each label and adding them together.

An ROC curve is a classification evaluation tool in deep learning. In real-world applications, some datasets have the problem of class imbalance. For example, a common case is that the number of negative images is larger than that of the positive images for medical datasets. A stable evaluation curve could be achieved by using the ROC curve. To summarize, the ROC curve has the following features: First, the curve can be used to check the impact of a specific threshold value on the generalization ability of a classifier. Second, the ROC can help determine the best threshold value, since the closer it is to the upper-left corner, the better the classifier is. Third, the ROC is a good tool to compare the performance of many different classifiers for each class intuitively.

In the figure of a typical ROC curve, the horizontal coordinate, i.e., false positive rate (*FPR*), and the vertical coordinate, i.e., true positive rate (*TPR*), are defined as follows:

(2) $$TPR=\frac{TP}{P}=\frac{TP}{TP+FN}.$$

(3) $$FPR=\frac{FP}{N}=\frac{FP}{TN+FP},$$

where *P* is the number of real positive samples and *N* is the number of real negative samples. *TP* means true positive, which is the positive samples that are predicted positively by the model. *FP* mans false positive, which is the negative samples that are predicted positively by the model; *FN* means false negative, which is the positive samples that are predicted negatively by the model; *TN* means true negative, which is the negative samples that are predicted negatively by the model. For a specified classifier, a pair of TPR and FPR points can be obtained according to the testing performance. As a result, this classifier can be mapped into a point on the ROC plain. The area under ROC curve (AUCROC) is used to quantify the classification ability, and a larger AUCROC indicates better classification performance.

There are three methods to calculate the AUC manually, the namely trapezoidal rule, the Mann–Whitney statistics [21], and the parameter rule. The first method uses the vertical line of each point on the x-axis and calculates the sum of small trapezoidal areas. The second method is proper for medical images, because it calculates the value of the possibility that positive samples are larger than the negative samples. The third method uses the mean and variance value when the samples obey a Gaussian distribution. In our work, these two functions roc_auc_score, roc_curve can be used by directly importing them from the *sklearn.metrics* library. After the AUC value is calculated, the performance of the classifier can be analyzed: (1) If AUC=1, the classifier is perfect. (2) If 0.5<AUC<1, the performance is better than guessing randomly. If a proper threshold value is set, the classifier can predict most of the cases correctly. (3) If AUC=0.5, the process of prediction is the same as a random guess, and there is no prediction value. (4) If AUC<0.5, it is worse than guessing. However, if predicting inversely, it is similar to the second case.

### 2.3. Visualization Using Class Activation Maps

Visualization of neural networks increases the interpretability of the networks in the field of computer vision. The complexity of medical images always makes the visualization harder. In our work, the class activation map (CAM) was used for visualization. The basic principle of the CAM is that it will produce a heat map of the input images, indicating the degree of similarity between the real class and the predicted class. Specifically, the technique used in this work was gradient-weighted class activation mapping (Grad-CAM) [22]. This method generates a localization map with the significant parts of the image highlighted by extracting the gradient of the classification target and letting the gradient flow into the last layer.

A convolutional neural network normally consists of a feature extractor, which is used to extract useful features, and a classifier, which classifies according to the extracted features. There are two kinds of classification models. One is feature extraction with flatten and softmax layers: A flatten layer is used to transform the three-dimensional images into one-dimensional vectors. A dense layer will then be added, and finally, there is a softmax function as the activation function for the output. The other is feature extraction with global average pooling (GAP) and softmax, where a global average pooling layer is used to substitute the flatten layer: this has the advantages of reducing the number of parameters, making the training process easy and preventing from overfitting. Based on the classification model, the CAM is generated.

For a traditional CNN model that has a flatten layer, if the last layer of the CNN has *n* feature maps, which means there are *n* weights for a neuron in the classifier layer and each neuron relates to a class, then the class activation map [22] for class *c* can be calculated as follows:

(4) $$L_{CAM}^{c}=\sum_{i=1}^{n}w_{i}^{c}A^{i},$$

where the weights for the *i*th neuron are: $w_{1}^{i}$, $w_{2}^{i}$, $w_{3}^{i}$, ⋯, $w_{n}^{i}$, and $A^{i}$ indicates the feature maps in the last layer. If a GAP is used to substitute the flatten layer, the classification score of class *c* [22] can be calculated as follows:

(5) $$S_{c}=\sum_{i=1}^{n}w_{i}^{c}GAP\left(A^{i}\right)=\frac{1}{Z}\sum_{i=1}^{n}\sum_{k=1}^{c1}\sum_{j=1}^{c2}A_{kj}^{i}w_{i}^{c},$$

where $w_{i}^{c}$ is the weight for the GAP and the size of a feature map is Z=c1∗c2. The value of $S_{c}$ is determined by the pixel value $A_{kj}^{i}$ and weights $w_{i}^{c}$. If the multiplication of the pixel value and weights is larger than 1, the sample will be classified into this current class *c*, and the model considers the original image as related to this class. This equation helps decide which part of the original image corresponds to a specific pixel.

CAMs are a very powerful tool for the visualization of the neural network’s decision-making process. However, they have certain limitations: (1) We can apply CAMs only if the CNN contains a GAP layer; (2) heat maps can be generated only for the last convolutional layer. To address these issues, gradient-weighted class activation mapping (Grad-CAM) is proposed. The class activation mapping for class *c* [22] can be generated by:

(6) $$L_{Grad-CAM}^{c}=\frac{1}{Z}\sum_{i=1}^{n}\sum_{k=1}^{c1}\sum_{j=1}^{c2}\frac{\partial S_{c}}{\partial A_{kj}^{i}}A^{i}.$$

Grad-CAM is the generalization of the CAM, and the gradient operator indicates the backpropagation. Grad-CAM was employed in our work due to its advantages. The code implementation included the following steps: (1) The output of the batch normalization (BN) layer [23] and the output of the whole network were extracted. (2) Backpropagation was computed from the output of the whole network to the output of the BN layer by using function *gradients* in *TensorFlow* to calculate the gradient automatically. (3) We used the gradients as the weights and multiplied them with the output of the BN layer. (4) Function *resize* in the *OpenCV* library was used to compound the feature maps to visualize.

## 3. Simulation Results

Our simulation process can be divided into three parts: (1) The raw data need to be preprocessed, including checking the data leakage, handling the class imbalance, performing the data augmentation, and generating new images. (2) The training process was conducted. (3) The testing and evaluation results showed the generalization ability of the model. Simulations were conducted on a GPU-equipped computer, using *TensorFlow* and *Keras*.

### 3.1. Data Preprocessing

The data used in our work were frontal-view chest X-ray images from patients. The whole dataset was obtained from https://nihcc.app.box.com/v/ChestXray-NIHCC (accessed on 10 Febuary 2021). Each image in the dataset includes 14 labels for 14 pathological conditions, such as consolidation, effusion, edema, atelectasis and so on. For each label, 1 means positive and 0 means negative. After classification, the pathological conditions can be utilized by physicians to detect eight different diseases. The original datasets were divided into three groups for training, validation, and testing, respectively.

Data leakage is a common problem for processing medical images, because one patient may have multiple images. Data leakage will lead to the overfitting problem, since it is difficult for the model to learn from similar features and to predict other new features. To ensure that there is no data leakage between any two datasets, the datasets should not contain the images from the same patient. The identification of unique patents of each set was collected by using the *set* function in Python, and then, the *intersection* function was used to check whether the two datasets contain information from the same patient.

Neural networks can only process the data in the format of float tensor. Therefore, formatting is important, since the original dataset contains images in PNG files. In *Keras*, there is a class named *ImageDataGenerator*, which can be used to finish the following tasks in sequence: read image files; encode the PNG files into RGB pixels; transform these pixels into a float tensor; scale the pixels in the range of [0,1]. Then, three generators are defined to load the images into the network. Several parameters can be set to proper values in *ImageDataGenerator*:

- Batch size. The batch size, the number of samples for one training, influences the optimization degree and speed. Since the network was trained on a GPU (2×Tesla V100)-equipped machine, batch_size=16 matches the GPU’s performance;
- Resolution. The original images provided in [24] have a size of 1024 × 1024, which is relatively too big to be processed. With the help of a Python generator in Keras, the images were scaled to 400 × 400, the value of which was chosen to balance the accuracy and learning speed.

**Figure 1:**
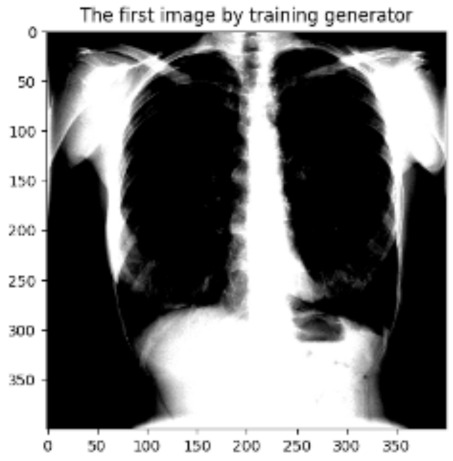
A chest X-ray image.

**Figure 2:**
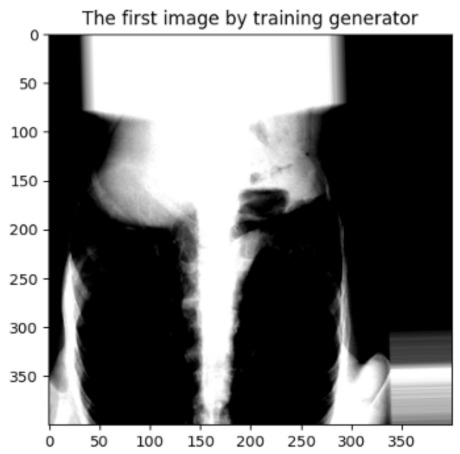
A chest X-ray image with data augmentation.

The data augmentation module was added to the generator, which means that the data were already augmented before feeding them into the neural network. In order to compare the image before and after data augmentation, the first image of the dataset is shown in Figure 1 by using the *plt.imshow* function. As shown in Figure 2, the image was shifted and zoomed after augmentation.

Class imbalance was handled by calculating the weight loss as the loss function. Specifically, for each label, the loss was weighted by the frequency of positive data $\left(w_{p}\right)$ and that of negative data $\left(w_{n}\right)$ as shown below:

(7) $$L\left(X,y\right)=\left\{\begin{array}{cc} w_{p}*\left(-log\left(Y=1|X\right)\right), & \;\mathrm{if}\;y=1,\\ w_{n}*\left(-log\left(Y=1|X\right)\right), & \;\mathrm{if}\;y=0, \end{array}\right.$$

where *Y* stands for predication and *X* means input labels.

### 3.2. Training

The pretrained network was used as the base model. A global pooling layer was added using function *GlobalAveragePooling2D*, and a fully connected layer was placed as the output layer by employing the Dense function with *Softmax* activation. In our work, the aim was to realize the classification of 14 pathological conditions, which is a multi-task classification problem. In this scenario, the effective activation was *Softmax*. The final output of the model is called the prediction, which is a 14-length vector with each element indicating the probability of a certain pathological condition. In order to compile the whole model, function *compile* was used, and several related parameters were set. For example, compiling the model required the type of loss function and the optimizer. The weighted loss was considered as the loss function, since the class imbalance problem was handled by the weighted loss. *Adam* was used as the optimizer since it has better performance than the traditional optimizers, such as the *Momentum* and *RMSprop* optimizers. Since “accuracy” was used as the metric, the accuracy of each training step and each validation step was displayed while running the code.

After all the preparations were completed, the network was trained by using training labels and images. The goal of the training was optimization, which means the model itself builds the connection of the output and output and learns the features. By using the fit_generator function, the model first fits the data to realize training and then performs the validation. Some parameters are important for the training and/or validation process:

- Steps per epoch means the number of steps for each epoch. Data in its batch size were the input from the generator to the network for each step. The relationship between this parameter and the batch size was (# steps per epoch) × batch size = # total training samples. Since the batch size was set to 16 because of the GPU performance and the total samples for training were 402, the steps per epoch should be 25;
- The value of the validation steps needs to be assigned, after the steps per epoch are determined. The validation steps were the total number of steps in the validation dataset. The validation steps should be two, since there were forty images for validation, and (# validation steps) × batch size = # total validation samples;
- The value of the epoch decides the total number of training samples. In each epoch, the network learns the features from all of the input images. In this work, the epoch was set to 80. The reason was that the plots with 80 epochs could clearly show the variation tendency of the accuracy and loss, and also, overfitting might occur if the network is trained for too many epochs. Early stopping was also used by stopping training if the accuracy did not increase for 10 epochs, which can help mitigate the overfitting problem to some extent.

When each epoch of training was finished, the weights of the current trained network were saved in a weight file, by calling the *model.save* function. The later training was based on the formerly saved weights.

The next step was to plot the loss curve for training and validation, which is useful for observing network convergence and the overfitting problem. Function *Matplotlib* in *Keras* was used for plotting. After all training and validation epochs, the loss for each epoch can be retrieved by calling the *history* function.

**Figure 3:**
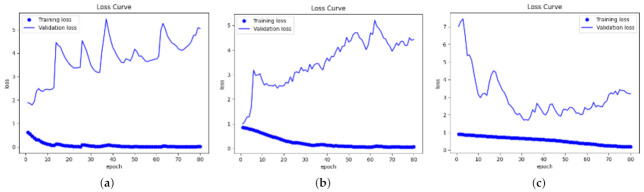
Loss curves for xx with/without DA. (**a**) DenseNet121 without DA. (**b**) DenseNet121 with DA. (**c**) ResNet50 with DA.

The training loss and validation loss of DenseNet121 without DA, DenseNet121 with DA, and ResNet50 with DA as the base model are shown in Figure 3. The results without DA and with DA were firstly compared. Figure 3a,b shows that both of the losses with or without DA for the training decreased from one to nearly zero with the increase of the epoch, while those for validation increased from one to almost five, which means that the model was overfit. Ideally, training loss and validation loss should have the same trends, if the model is well fit. The figures also demonstrate that the model with DA had better performance than that without DA. The figures show that the model with DA learned the model slower than that without DA, since more images needed to be fed into the the network after data augmentation. The curves for DenseNet121 without DA fluctuated more than those with DA. The loss curves by using ResNet50 as the base model with DA are also presented. Compared with DenseNet121, ResNet50 took more time to train because the training loss converged at around the 70th epoch.

The training accuracy of using these three models is shown in Table 1. DenseNet121 with DA had the highest training accuracy, followed by DenseNet121 without DA and then ResNet50. The reason was that the dataset became larger and more diversified after DA, and thus, the network was trained to be optimal. ResNet50 had the lowest training accuracy, since there were fewer shortcut connections inside of the base model, and consequently, the learning ability was poorer.

### 3.3. Testing and Evaluation

All the testing images were fed into the model, and the prediction results could be obtained. To test the network, function *predict_generator* was used as the major function. The output of this function was a list, which included the probability of classification for each label. When this probability was larger than the threshold value of 0.5, the program considered the prediction as correct. After comparing the prediction results with the real label of each image, the generalization ability of the model could be known with the self-defined function to calculate the testing accuracy. The classification accuracy for testing the datasets using DenseNet121 without DA, DenseNet121 with DA, and ResNet50 with DA is shown in Table 2. This table shows that DenseNet121 had better performance than ResNet50, and DA was beneficial for improving the classification accuracy.

**Figure 4:**
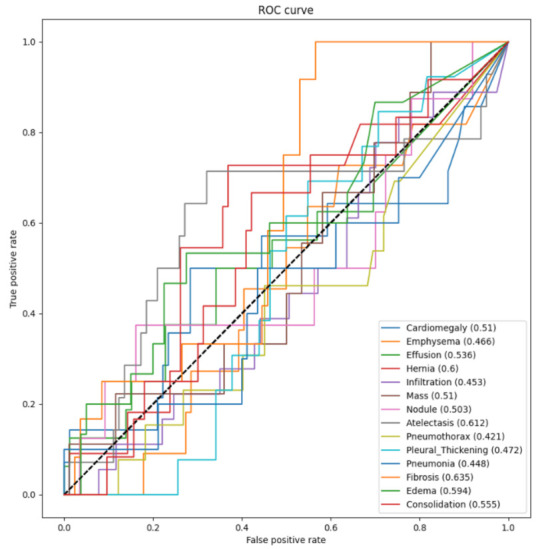
The ROC and AUCROC for DenseNet121 without DA.

In order to evaluate the model, the receiver operating characteristic (ROC) curves were generated, and the area under the curve (AUC) was calculated. *Keras* has a library, *sklearn*, which can conduct some advanced computations in machine learning and computer vision. For the evaluation, functions roc_auc_score and roc_curve were imported from the library to calculate the AUCROC and to derive the ROC curve. Figure 4 illustrates the ROC curves and the AUCROC values of DenseNet121 without DA for the 14 pathological conditions. The horizontal axis indicates the false positive rate, while the vertical axis indicates the true positive rate. The AUCROC score for each class is listed at the lower-right corner of this figure, e.g., for cardiomegaly, the AUCROC was 0.51, which means that the area under curve for the label was 0.51. The figure shows that the ROC curves for several pathologies lie below the straight line that passes through points (0,0) and (1,1). For these pathologies, the classifier worked even worse than random guessing. The AUCROC values of five pathologies, i.e., emphysema, infiltration, pneumothorax, pleural thickening, and pneumonia, were all less than 0.5, which means that the classifier could not diagnose most of the images in these classes correctly. Therefore, this figure indicates that the classification ability of DenseNet121 without DA was relatively poor.

**Figure 5:**
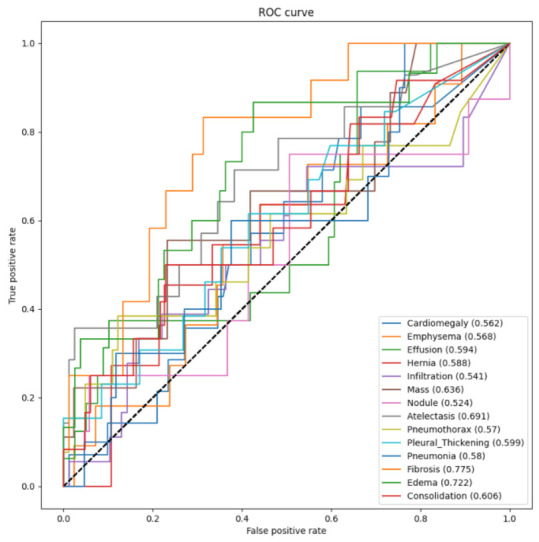
The ROC and AUCROC for DenseNet121 with DA.

Figure 5 illustrates the ROC curves and the AUCROC values of DenseNet121 with DA for the 14 pathological conditions. This figure shows that most of these ROC curves are located above the dotted line that passes through points (0,0) and (1,1), and all of the AUCROC values are larger than 0.5. The reason was that the images were preprocessed with DA, which led to a better-trained network. For fibrosis, the ROC curve lies significantly higher than the other curves and is mostly close to the upper-left corner, and its AUCROC was the largest with a value of 0.775, which means that its classifier had the best performance among all 14 classifiers. For nodule and infiltration, their AUCROC values were just slightly larger than 0.5, which means that these classifiers could help predict these pathological conditions, but the performance was relatively poor.

**Figure 6:**
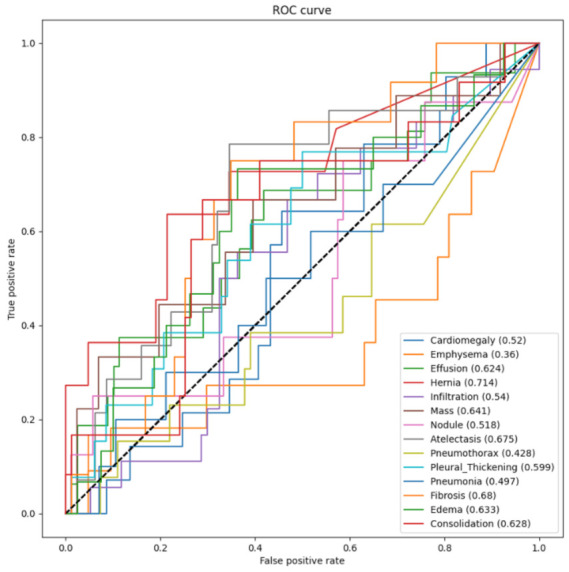
The ROC and AUCROC for ResNet50 with DA.

Figure 6 illustrates the ROC curves and the AUCROC values of ResNet50 with DA for the 14 pathological conditions. Compared to Figure 5, more ROC curves using ResNet50 with DA lie below the straight dotted line that passes through points (0,0) and (1,1) than those using DenseNet121 with DA. The largest AUCROC value was for fibrosis, with the value of 0.68, which was smaller than that of using DenseNet121 with DA. The AUCROC values of three classes, i.e., emphysema, pneumothorax, and pneumonia, were smaller than 0.5, which means that these classifiers could not help predict these pathological conditions.

The comparison of the ROC curves and AUCROC values for different networks demonstrated that the classifiers trained by DenseNet121 had better performance than those trained by ResNet50. The results also indicated that DA improved the classification capability for all of the classes. Most of the ROC curves lie above the straight dotted line that passes through points (0,0) and (1,1), but they are not close to the upper-left corner enough, because the dataset used for testing was relatively small.

### 3.4. Visualization

**Figure 7:**
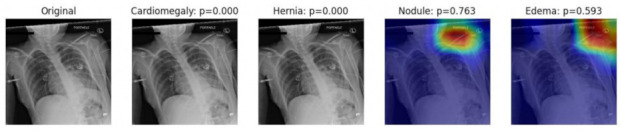
Visualization of the diagnosis heat maps of one image example by the use of Grad-CAM.

**Figure 8:**
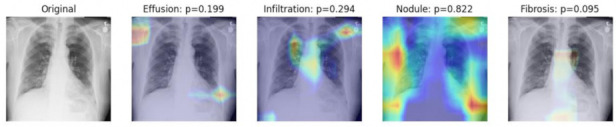
Visualization of the diagnosis heat maps of the second example by the use of Grad-CAM.

The visual explanation of classification decision-making was produced by using Grad-CAM techniques. The heat maps of using DenseNet121 as the base model are shown in Figure 7 and Figure 8. These chest X-rays were randomly selected from the datasets, and only the four most probable diagnosis heat maps are shown in the figure. The probability of diagnosing a certain pathological condition is demonstrated in each of the subfigures. For example, in Figure 7, the original chest X-ray image is shown in the first subfigure. The second and third subfigures indicate that it is impossible for the image to be classified as cardiomegaly or hernia. The fourth and fifth subfigures mean that the image has a probability of 0.763 and 0.593 to be diagnosed as nodule and edema, respectively. Figure 8 shows that the original image has the possibility of being diagnosed into four pathological conditions, and the most probable one is nodule with a probability of 0.822.

## 4. Conclusions

A deep learning approach was proposed to use transfer learning and pretrained networks to recognize and classify chest X-ray images into 14 pathological conditions, and therefore help with diagnosing diseases related to these pathological conditions. The performance of the two adopted pretrained networks DenseNet121 and ResNet50 was compared, and DA was also used to further improve the performance. Evaluation metrics, such as the accuracy, ROC curves, and AUCROC curves were utilized. The simulation results showed that the network using DenseNet121 as the base model with DA had a better generalization ability on the testing datasets. In the future, multiple transfer learning methods could be used together with ensemble classifiers to further improve the performance of the proposed work. The potential use of the other datasets, such as PadChest, ChexPpert, and MIMIC-CXR, will be explored in our future work.

## Figures and Tables

**Table 1:** Training accuracy for different networks.

Networks	Type of Data Processing	Training Accuracy
DenseNet121	Without data augmentation	0.89
	With data augmentation	0.92
ResNet50	With data augmentation	0.84

**Table 2:** Testing accuracy for different networks.

Networks	Type of Data Processing	Testing Accuracy
DenseNet121	Without data augmentation	0.82
	With data augmentation	0.84
ResNet50	With data augmentation	0.76

## Data Availability

The data analyzed in this study are openly available at https://nihcc.app.box.com/v/ChestXray-NIHCC accessed on 10 Febuary 2021.
